# Efficacy and safety of acupuncture on childhood attention deficit hyperactivity disorder: A protocol for systematic review and meta-analysis

**DOI:** 10.1097/MD.0000000000023953

**Published:** 2021-02-05

**Authors:** Yong Lin, Hongjiao Jin, Bo Huang, Ning Zhao, Zhu Li, Jiao Mao, Changda Chen, Jie Xu, Jun Zhang, Biqin Shuai

**Affiliations:** The First People's Hospital of Zunyi (The Third Affiliated Hospital of Zunyi Medical University), No. 98 Fenghuang Road, Huichuan District, Zunyi, Guizhou, China.

**Keywords:** acupuncture, attention deficit hyperactivity disorder, protocol, systematic review

## Abstract

**Introduction:**

The purpose of this paper is to evaluate the efficacy and safety of acupuncture in the treatment of childhood attention deficit hyperactivity disorder (ADHD).

**Methods and analysis:**

We will electronically search PubMed, Medline, Embase, Web of Science, the Cochrane Central Register of Controlled Trial, China National Knowledge Infrastructure, China Biomedical Literature Database, China Science Journal Database, and Wan-fang Database from their inception. Also, we will manually retrieve other resources, including reference lists of identified publications, conference articles, and grey literature. The clinical randomized controlled trials or quasi-randomized controlled trials related to acupuncture treating pediatric ADHD will be included in the study. The language is limited to Chinese and English. Research selection, data extraction, and research quality assessment will be independently completed by 2 researchers. Data were synthesized by using a fixed effect model or random effect model depend on the heterogeneity test. The scores of Revised Conners’ Parent Rating Scale (CPRS-R), Conners Teacher Rating Scale (CTRS-R), and Child Behavior Checklist (CBCL) will be the primary outcomes. Besides, the scores of the Conners Continuous Performance Test, Internal Restlessness Scale, and Behavior Assessment System for Children (BASC), and the possible adverse events will also be assessed as secondary outcomes. RevMan V.5.3 statistical software will be used for meta-analysis, and the level of evidence will be assessed by Grading of Recommendations Assessment, Development, and Evaluation (GRADE). Continuous data will be expressed in the form of weighted mean difference or standardized mean difference with 95% confidence intervals (CIs), while dichotomous data will be expressed in the form of relative risk with 95% CIs.

**Ethics and dissemination:**

The protocol of this systematic review (SR) does not require ethical approval because it does not involve humans. We will publish this article in peer-reviewed journals and presented at relevant conferences.

**Systematic review registration:**

OSF Registries, DOI: 10.17605/OSF.IO/XVYP9 (https://osf.io/xvyp9)

## 1 Introduction

Attention deficit hyperactivity disorder (ADHD) mainly manifests as inattention, and/or hyperactivity and impulsivity^[1]^; these behaviors appear on children under 12, and continues to adulthood^[2]^; it affects 2.2% to 17.8% of children and youth, being the most common neurodevelopmental disorder.^[3]^ Among children with ADHD, approximately one-third persists to adulthood.^[4]^

In the treatment of ADHD, pharmacological therapies, including methylphenidate, amphetamine, atomoxetine, and guanfacine, were reported to be effective for some features,^[5]^ but failed to improve mood, self-esteem, and social relationship^[6]^; besides, ADHD patients showed low medicine compliance after 1 year.^[7]^ Nonpharmacological treatments, including behavior management approaches (parent training, classroom management, and peer interventions), cognitive training, motivational enhancement, academic, organizational, social skills training, and neurofeedback^[8]^ are also considered as valid options.^[9–12]^

Meanwhile, a wide range of complementary and alternative medicines are used; a study showed that 12% to 64% of ADHD patients received complementary and alternative medicine approaches, including nutritional intervention, herbal supplements, acupuncture, massage, and yoga.^[13–15]^ As one of the most common form of complementary and alternative medicine, acupuncture is widely applied to treat various of conditions,^[16]^ it is reported to improve attention and school performances on children,^[17,18]^ and it is popular in treating ADHD in Asian countries.^[19]^ Till today, it is still uncertain if acupuncture could improve the core symptoms in ADHD; thus, to access the safety and efficacy of acupuncture in the treatment of ADHD, we plan to conduct this systematic review and meta-analysis, hoping to provide further reference for future clinical practice.

## 2 Methods

The protocol has been registered on OSF as Registration DOI: 10.17605/OSF.IO/XVYP9 (https://osf.io/ha97r). The protocol follows the Preferred Reporting Items for Systematic Reviews and Meta-Analyses Protocols (PRISMA-P) 2015 statement guidelines.^[20]^ We will report the changes in the full review if necessary.

### 2.1 Inclusion and exclusion criteria for study selection

#### 2.1.1 Inclusion criteria

This study will include randomized controlled trials (RCTs) of acupuncture for ADHD children, whether using blind method or allocation concealment method, including those using a quasi-random method such as alternate allocation or allocation by birth date. We included both parallel and crossover studies. The language of the trials to be included should be English or Chinese.

#### 2.1.2 Exclusion criteria

Following studies would be excluded: case reports and reviews, literature not in English or Chinese language, and clinical research studies that compared different kinds of acupuncture.

### 2.2 Types of participants

We would include patients with ADHD under the age of 18 years, regardless of gender or race, diagnosed by standard criteria such as the Diagnostic and Statistical Manual of Mental Disorders (DSM) or the International Classification of Diseases (ICD).

### 2.3 Types of interventions and comparators

Acupuncture for treating ADHD include traditional acupuncture, electro-acupuncture, laser acupuncture, auricular acupuncture, etc. These interventions can be used alone or in combination. The details of acupuncture treatment should be clearly illustrated according to STRICTRA,^[21]^ involving the needle selection, acupoints selection, manipulations, course, etc. Controlled interventions include no intervention, placebo acupuncture or sham acupuncture; standard treatments (Western medicine, herbal medicine, or combination of both); or other non-acupuncture interventions.

### 2.4 Types of outcome measures

#### 2.4.1 Primary outcomes

We select the score of Revised Conners’ Parent Rating Scale (CPRS-R), Conners Teacher Rating Scale (CTRS-R), and Child Behavior Checklist (CBCL) as primary outcomes.

#### 2.4.2 Secondary outcomes

We also care about the scores of the Conners Continuous Performance Test, Internal Restlessness Scale, and Behavior Assessment System for Children (BASC); meanwhile, the possible adverse effects are also taken into consideration, including fainting, stuck needle, bent needle, broken needle, and hematoma.

## 3 Data sources

### 3.1 Electronic searches

Following databases will be searched: PubMed, Web of Science, the Cochrane Central Register of Controlled Trials, AMED, MEDLINE, EMBASE, Cochrane Library, China National Knowledge Infrastructure (CNKI), Wanfang data, Chinese Scientific Journals Database (VIP), and China biomedical literature database (CBM). We will select the eligible studies published up to October 31, 2020. The search terms used in the systematic review are as follows: acupuncture, attention deficit hyperactivity disorder.

We will not apply any language, population or national restrictions. The specific search strategy will be (taking PubMed as an example) listed on Table 1. Similar search strategy will be applied to other electronic databases.

**Table 1:** PubMed search strategy.

#1	childhood attention deficit hyperactivity disorder[MeSH Terms]
#2	childhood attention deficit hyperactivity disorder[Text Word]
#3	pediatric ADHD[MeSH Terms]
#4	pediatric ADHD[Text Word]
#5	or/#1–4
#6	acupuncture[MeSH Terms]
#7	acupuncture[Text Word]
#8	acupuncture therapy[MeSH Terms]
#9	acupuncture points[MeSH Terms]
#10	electroacupuncture[MeSH Terms]
#11	moxibustion[MeSH Terms]
#12	moxibustion[Text Word]
#13	acupressure[Text Word]) OR moxibustion[Text Word]
#14	or/#6–13
#15	#5 and #14

We will identify relevant RCTs and the selected studies will be analyzed according to the Cochrane Handbook.

### 3.2 Searching other resources

**Figure 1:**
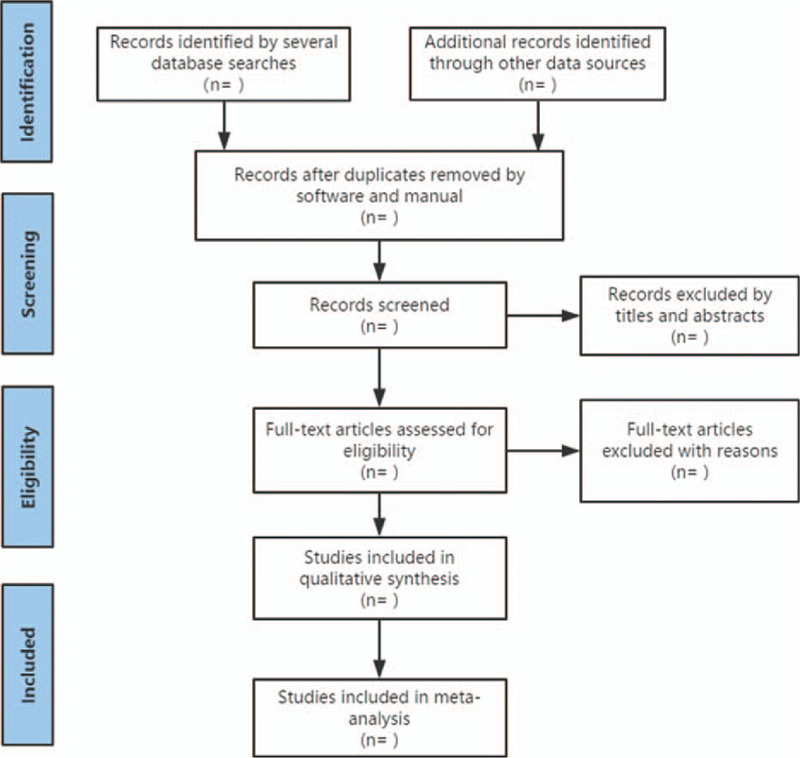
The research flow chart.

We also retrieve manual related documents, such as replacing and supplementing some reference documents, medical textbooks, clinical laboratory manuals, and the World Health Organization International Registry of Clinical Trials (ICTRP). At the same time, we will contact experts and authors in this field to obtain important information that cannot be found in the search. The research flow chart is shown in Figure 1.

## 4 Data collection and analysis

### 4.1 Selection of studies

Two independent researchers (LY and JH) will assess the full-text articles from the search results independently against the inclusion and exclusion criteria. Discrepancies will be discussed and resolved by consensus with a third author (HB).

### 4.2 Data extraction and management

The following information will be extracted from each study: research number, data extractor, date of data extraction, general situation of the study, research methodology, research population, baseline comparability, interventions, main outcome indicators, secondary outcome indicators, combined drug use, adverse reactions or complications, etc. For those with questions or incomplete information, we will try to contact the author to obtain information before deciding whether to include it.

### 4.3 Assessment of the reporting quality and risk of bias

Two of the authors (LY and JH) individually assessed the risk of bias using assessments included in the study were evaluated in the Cochrane System Evaluator's Manual for RCT quality evaluation criteria. Assessing the risk of bias: random sequence generation; allocation concealment; blinding of participants and personnel; blinding of outcome assessment; incomplete outcome data; selective outcome reporting; other bias. Every domain was classified as high risk of bias, low risk of bias, or unclear risk of bias. Any arising difference was resolved by discussion.

### 4.4 Measures of a treatment effect

We will measure continuous data with mean difference (MD) or standard MD (SMD) for the therapeutic effect with 95% CIs. For dichotomous data, risk ratios (RRs) with 95% CIs will be calculated.

### 4.5 Management of missing data

To obtain the missing data, we will contact the corresponding author. If no response will be obtained, we will analyze only the available data and describe the reason and impact of this exclusion in the paper.

### 4.6 Assessment of a reporting bias

Publication bias will be explored through funnel plot analysis. GRADE profiler 3.6 is used to evaluate the quality of evidence. The specific contents include limitations of research, inconsistency of research results, indirect evidence, inadequate accuracy, publication bias. Finally, the quality of evidence is divided into 4 levels: high-level evidence, intermediate evidence, low-level evidence, and very low-level evidence.

### 4.7 Assessment of heterogeneity

All literature will use *I*^2^ value of the Chi-squared test (a = 0.1) to determine the heterogeneity. When *I*^2^ ≤50%, it is considered acceptable. When *I*^2^ > 50%, subgroup analysis should be performed to identify potential causes and record them.

### 4.8 Data synthesis and grading of quality of evidence

RevMan 5.3 software was used for statistical analysis of data. RR was used for binary variables and MD was used for continuous variables. Heterogeneity analysis will be conducted by heterogeneity test; *P* and *I*^2^ represent the size of heterogeneity among multiple studies. When *P* is greater than .1 and *I*^2^ is less than 50%, it suggests heterogeneity is small; on the contrary, it suggests heterogeneity is large. Heterogeneity is mainly handled by subgroup analysis. Sensitivity analysis is used to test the reliability of the overall effect.

### 4.9 Subgroup analysis

When the heterogeneity test results are heterogeneous, we will conduct subgroup analysis to explore the possible causes of heterogeneity. The effects of different types of acupuncture therapy including design scheme, severity of illness, age, sex, and mild or severe AP were analyzed. We will also delete low-quality and/or medium-quality studies to check the robustness of the results.

### 4.10 Sensitivity analysis

Sensitivity analysis will be used to test the quality of the research contained in the sampled documents. The stability of the conclusions can be tested by reanalyzing the conclusions by inputting missing data and changing the type of research.

### 4.11 Ethics and dissemination

The results of the system review will be published in peer-reviewed journals, disseminated at relevant meetings, or disseminated in peer-reviewed publications, and we use aggregated published data to exclude individual patient data, so ethical approval, and informed consent are not required.

## 5 Discussion

ADHD is a multidimensional chronic neurodevelopmental condition that affects 8.4% of U.S. children between 2 and 17-years-old, when untreated, it may become a long-term condition into the adulthood.^[22]^ In children, ADHD mainly presents executive impairments in inhibitory and working memory^[23]^; pragmatics^[24]^; emotional dysregulation, including emotional inflexibility, slow return to emotional baseline, and low threshold for emotional excitability^[25]^; sensory integration deficits,^[26]^ including visual motor integration and time perception deficits; and motor performance issues.^[27]^

On children with ADHD, the goals of treatment are symptom reduction, social and cognitive function improvement.^[22]^ Along with medication and behavior therapy, complementary and alternative medicine (CAM) is widely applied in the treatment of ADHD, between 12% and 64% ADHD children received CAM, including nutritional interventions, electroencephalographic biofeedback, herbal and natural health products, massage and yoga, etc.^[15]^ As a major form of CAM, acupuncture is reported benefit for ADHD children and being prevalent in the management of this condition.

A decade ago, due to the limited RCTs evidence, former systematic reviews failed to conclude the efficacy and safety of acupuncture in the treatment of ADHD.^[28,29]^ Recent studies offered more refences on this topic. A statistically significant greater reduction in attention deficit and hyperactivity symptoms was observed on children who received auricular therapy on acupoints, comparing with those who received sham acupoints.^[30]^ Besides, a pilot study of RCT suggested a trend of greater improvement on ADHD scores on children received acupuncture, and no reserve event was reported.^[31]^ Furthermore, the combination of scalp acupuncture and EEG biofeedback achieves the superior efficacy on children with ADHD as compared with the EEG biofeedback therapy alone.^[32]^ Moreover, on ADHD children, a complementary and alternative medicine combination, including acupuncture, applied kinesiology, and respiratory exercises improved sleeping conditions rapidly, as well as hand writing.^[33]^ Another study showed that combining with behavior therapy, acupuncture had a positive effect on reducing ADHD symptoms on preschool children.^[34]^ Comparing to routine western medicine, acupuncture showed a better effect on improving mild brain dysfunction on children.^[35]^

As a result, to further investigate the safety and efficacy of acupuncture in the treatment of childhood ADHD, we would like to conduct this systematic review and meta-analysis, this work might contribute to the further evaluation of acupuncture treatment.

## Author contributions

**Conceptualization:** Yong Lin, Hongjiao Jin.

**Data curation:** Ning Zhao, Zhu Li.

**Formal analysis:** Hongjiao Jin, Ning Zhao.

**Funding acquisition:** Bo Huang.

**Methodology:** Hongjiao Jin, Jie Xu.

**Project administration:** Bo Huang, Yong Lin.

**Software:** Jiao Mao, Changda Chen.

**Supervision:** Bo Huang.

**Validation:** Jie Xu, Jun Zhang.

**Visualization:** Jun Zhang, Biqin Shuai.

**Writing – original draft:** Yong Lin, Hongjiao Jin.

**Writing – review & editing:** BO Huang, Biqin Shuai.
